# Limited Utility of Circulating Cell-Free DNA Integrity as a Diagnostic Tool for Differentiating Between Malignant and Benign Thyroid Nodules With Indeterminate Cytology (Bethesda Category III)

**DOI:** 10.3389/fonc.2019.00905

**Published:** 2019-09-18

**Authors:** Shilpa Thakur, Andrew Tobey, Brianna Daley, Sungyoung Auh, Mary Walter, Dhaval Patel, Naris Nilubol, Electron Kebebew, Aneeta Patel, Kirk Jensen, Vasyl Vasko, Joanna Klubo-Gwiezdzinska

**Affiliations:** ^1^Metabolic Disease Branch, National Institute of Diabetes and Digestive and Kidney Diseases, National Institutes of Health, Bethesda, MD, United States; ^2^National Institute of Diabetes and Digestive and Kidney Diseases, National Institutes of Health, Bethesda, MD, United States; ^3^Core for Clinical Laboratory Services, National Institute of Diabetes and Digestive and Kidney Diseases, National Institutes of Health, Bethesda, MD, United States; ^4^Cancer Center – Froedtert Hospital, Milwaukee, WI, United States; ^5^Endocrine Oncology Branch, National Cancer Institute, National Institutes of Health, Bethesda, MD, United States; ^6^Division of General Surgery, Endocrine Oncology Research Laboratory, Department of Surgery, Stanford Medicine, School of Medicine, Stanford, CA, United States; ^7^Department of Pediatric Endocrinology, Uniformed Services University of the Health Sciences, Bethesda, MD, United States

**Keywords:** thyroid cancer, AUS/FLUS, circulating cell free DNA, integrity, liquid biopsy

## Abstract

**Background:** Analysis of plasma circulating cell-free DNA integrity (cfDI) has emerged as a promising tool in the diagnosis of malignant vs. benign tumors. There is limited data on the role of cfDI in thyroid cancer. The goal of this study was to analyze cfDI as a biomarker of malignancy in patients with cytologically indeterminate thyroid nodules.

**Methods:** The cfDI was measured in the plasma of patients with cytologically indeterminate thyroid nodules. All patients underwent plasma collection within 24–72 h before surgical treatment for thyroid nodules. Additionally, samples were collected from seven patients via the vein draining the thyroid and peripheral vein during surgery. Quantitative real-time PCR was performed on the isolated cell-free DNA using two different primer sets (115 and 247 bp) to amplify consensus ALU sequences. The cfDI was calculated as the ratio of ALU247 to ALU115.

**Results:** All data are given as median [25th−75th percentile]. The study group consisted of 67 patients with 100 nodules, 80.6% (54/67) women, aged 43 [33-60] years. There was no difference in cfDI between 29 patients with benign nodules (0.49 [0.41–0.59]) and 38 patients with malignant lesions (0.45 [0.36–0.57], *p* = 0.19). There was no difference in cfDI in the vein draining the thyroid (0.47 [0.24–1.05]) and peripheral vein (0.48 [0.36–0.56], *p* = 0.44). In comparison to thyroid cancer patients, patients with benign nodules were characterized by significantly higher concentrations of ALU115 (1,064 [529–2,960] vs. 411 [27–1,049] ng/ml; *p* = 0.002) and ALU247 (548 [276–1,894] vs. 170 [17-540] ng/ml; *p* = 0.0005), most likely because benign tumors were larger (3, [1.8–4.1 cm]) than malignant lesions (0.7 [0.23–1.45], *p* < 0.0001). Women had significantly lower cfDI (0.45 [0.27–0.54]) than men (0.56 [0.44–0.8], *p* = 0.011).

**Conclusion:** The cfDI measured in the vein draining the thyroid is similar to the cfDI measured in the antecubital vein, validating cfDI measurements by peripheral liquid biopsy. Analysis of cfDI needs to be stratified by patients gender. In contrast to its diagnostic utility in aggressive cancers, cfDI has limited utility as a biomarker of malignancy in cytologically indeterminate thyroid nodules.

## Introduction

Thyroid nodules are frequently identified in clinical practice. In the United States, nearly 4–7% of adults have been identified as having thyroid nodules by palpation, while high resolution ultrasonography has led to the detection of thyroid nodules in around 20–76% of the general population (1). Ultrasound guided-Fine Needle Aspiration Biopsies (US-FNAB) have suggested that only 5–10% of thyroid nodules are malignant in nature (2). At present, US-FNAB is the most effective method for the diagnosis of thyroid cancer. Moreover, it helps in stratifying thyroid nodules into six diagnostic categories per The Bethesda System for Reporting Thyroid Cytopathology (TBSRTC): Bethesda I, Non-diagnostic or Unsatisfactory; Bethesda II, Benign; Bethesda III, Atypia of Undetermined Significance or Follicular Lesion of Undetermined Significance (AUS/FLUS); Bethesda IV, Follicular Neoplasm or Suspicious for a Follicular Neoplasm (FN/SFN); Bethesda V, Suspicious for Malignancy (SUP); Bethesda VI, Malignant.

Each of these diagnostic categories are associated with a certain risk of malignancy and, as such, certain management guidelines are recommended (3, 4). Bethesda I and II categories are associated with very low-risk of malignancy (0–10%) and therefore, surgery is not routinely recommended in these patients. On the contrary, Bethesda V and VI fall under high-risk categories (45–96% risk of malignancy) and consequently, either lobectomy or total-thyroidectomy is the most commonly applied treatment option. The remaining two categories—Bethesda III and IV—are considered intermediate-risk categories (6–40% risk of malignancy) and pose clinical management challenges (5). Among all patients diagnosed with thyroid nodules, nearly 30% have indeterminate cytology. There are three usual management options for patients with Bethesda III and IV nodules: (1) to repeat FNAB and/or (2) to perform molecular testing or (3) perform a diagnostic lobectomy. However, hemi- or total-thyroidectomy in a patient with benign nodules is an excessive treatment, whereas performing lobectomy in patients with malignant tumors characterized by higher risk features, such as extrathyroidal extension or lymph node metastases, can be seen as under-treatment (6).

At present, there are several commercially available molecular tests including, but not limited to, Afirma v2 by Veracyte, ThyroSeq v3 by CBLPath and University of Pittsburgh Medical Center and ThyGenX/ThyraMIR by Interpace Diagnostics (7). While these tests have shown reasonable performance when tested on single institutional cohorts, the variation in performance has been observed for most of these tests when evaluated in different independent cohorts (7, 8). Moreover, these tests are expensive, posing another challenge in the medical industry. This emphasizes the need for alternative methods that should not only be reliable and sensitive but should be inexpensive as well.

Among others, one possible alternative could be a liquid biopsy consisting of the analysis of circulating cell-free DNA integrity (cfDI). The main advantage of this method is that it is less invasive, involving only a peripheral blood sample. Over the last two decades, analysis of cfDI has emerged as a promising biomarker in cancer diagnosis and prognosis (9, 10). The cfDI has been observed to be increased in various cancers (11). DNA integrity is calculated as the ratio of the concentration of longer DNA fragments to shorter fragments in the plasma or serum. The short DNA fragments that are nearly 180–200 bp in size are shed in the blood by normal cells during apoptosis. Unlike normal cells, tumor cells release longer DNA fragments in the blood primarily through necrosis or autophagy (12). This release of longer fragments by tumor cells causes an increase in DNA integrity in cancer patients. Analysis of cfDI has been proven to be effective in differentiating malignant from benign nodules in hepatocellular carcinoma, breast, colorectal and prostate cancer patients (13–16).

In this study, we aimed to investigate if cfDI could serve as a diagnostic tool in differentiating benign from malignant lesions in patients with thyroid nodules with indeterminate cytology (Bethesda Category III).

## Materials and Methods

### Study Design

We conducted a cohort study of 67 patients with indeterminate thyroid nodules (Bethesda III) who underwent hemi- or total-thyroidectomy. None of the patients had known active malignancies in organs other than the thyroid. All patients underwent 5 ml of blood collection within 24–72 h before surgery. The blood samples were centrifuged and processed immediately after collection and plasma was stored at −80°C. Additionally, samples were collected from seven patients via the vein draining the thyroid and peripheral antecubital vein during surgery. The final pathology diagnosis of the thyroid nodules was obtained after surgery. The subset of pathology tissue samples was subjected to Ion Torrent™ Oncomine™ Comprehensive Assay v3 (OCAv3) next-generation sequencing, analyzing single nucleotide variants (SNV), small insertions and deletions (INDEL), copy number variants (CNV) and gene fusions (GF) from 161 cancer driver genes.

This study was approved by the NIDDK Institutional Review Board (NCT00001160) and the consent has been obtained from each patient.

### Cell-Free DNA Extraction

All samples were subjected to DNA extraction. The isolation of circulating cell-free DNA (cfDNA) was performed on the KingFisher™ Duo Prime particle processor (ThermoFisher Scienific) using the MagMax Cell Free DNA isolation kit (cat# A29319). The magnetic bead-based purification format enables for processing of as low as 600 μl of plasma. For samples where plasma volume was less than 600 μl, PBS was added to 600 μl and corrected for the dilution factor to obtain a uniform 600 ul cfDNA extraction volume for the whole group. The purified cfDNA sample was eluted in 30 μL volume.

### Quantitative PCR of ALU Repeats

The isolated cfDNA was subjected to quantitative real-time PCR, which used two different primer sets to amplify both shorter (ALU115) and longer (ALU247) fragments of consensus ALU sequences. Previously published primer sequences were used to amplify both ALU115 and ALU247 fragments (13). The standard PCR reaction mixture in each well contained 10μL iQ SYBR Green Supermix (Bio-Rad), 1 μL each of forward and reverse primers, 1 μL isolated cell-free DNA and 7 μL of RNase free water for a total reaction volume of 20 μL. The samples were run along with standards on the StepOnePlus Real-Time PCR Detection System (Applied Biosystems). The Human genomic DNA (G3041, Promega) was used as a standard to determine ALU247 and ALU115 amplicons concentration. The absolute concentration of ALU247 and ALU115 fragments in each sample was determined from the standard curve (Supplemental Figure 1). The assay was repeated twice in two technical replicates and the cell-free DNA integrity index (cfDI) was calculated as the ratio of the concentration of ALU247 fragments to ALU115 fragments.

### Statistical Analysis

All statistical analyses were performed using GraphPad Prism software (version 7.4) except, multiple linear regression analysis. An unpaired *t*-test was performed for normally distributed variables and for analysis of subset of patients with plasma samples derived from the vein draining the thyroid and peripheral vein, a paired *t*-test was performed. In case of skewed distribution, the Mann-Whitney test was used for comparison between two groups. For qualitative variables, the Chi-square test was used. Data are expressed as median and 25–75% inter-quartile ranges [IQR]. The Pearson correlation coefficient was used to test for a correlation between two continuous variables. Multiple linear regression model was used for each of cfDI, ALU115, and ALU247 as a dependent variable with dichotomized age (<55 or ≥ 55), dichotomized tumor size (≤ 2 or >2) and gender as independent variables. This regression analysis was conducted using SAS Version 9.4 (SAS Institute, Cary, NC). All tests were two-tailed and *p*-values less than 0.05 were considered significant.

## Results

### Patient Characteristics

The study cohort consisted of 67 patients with 100 thyroid nodules. Of these, 80.6% were women aged 43; median age was 43[33-60] years. The median maximum diameter of a thyroid nodule based on ultrasound measurements was 1.9 cm [1–3.3] and the median volume was 1.48 cm^3^ [0.48–6.35]. All subjects underwent surgical treatment for thyroid nodules. The final pathology revealed a thyroid cancer diagnosis in 38 patients, including 20 patients with micro-papillary thyroid cancer (micro-PTC), while the remaining 29 patients had benign adenomatoid nodules. The age of the patients was normally distributed in both cancer and benign groups, with no statistical difference between the groups (Table 1). No statistical difference was observed in the gender distribution between the groups (Table 1). Patients with benign nodules were characterized by significantly larger tumor size (3 cm [1.8–4.1]) in comparison to cancer patients (0.7 cm [0.3–1.4], *p* < 0.001) (Table 1), likely due to large proportion of micro-PTC in our cohort of cancer patients.

**Table 1:** Baseline characteristics of the patients included in the study.

**Baseline characteristics**	**Overall (*N* = 67)**	**Benign nodules (*N* = 29)**	**Thyroid cancer (*N* = 38)**	***P*-value**
Age at diagnosis (median, 25–75% IQR) years	43 (33–60)	43 (32–60)	44 (33–61)	0.44
Gender (no of patients, %)				0.99
Male	13 (19.4%)	6 (20.7%)	7 (18.4%)	
Female	54 (80.6%)	23 (79.3%)	31 (81.6%)	
Tumor size (median, 25–75% IQR) cm	1.1 (0.45–2.75)	3 (1.8–4.1)	0.7 (0.23–1.45)	<0.0001^*^
Presence of pathogenic mutations ^#^				0.02^*^
Present	22 (53.7%)	4 (28.6%)	18 (67%)	
Absent	17 (41.4%)	10 (71.4%)	7 (25.9%)^##^	
Glomerular filtration rate (median 25–75%IQR) ml/1.73m^2^/min	96 (60–107)	106 (96–124)	108 (89–119)	0.32
Creatinine (median, 25–75% IQR) mg/dl	0.69 (0.61–0.80)	0.72 (0.63–0.78)	0.68 (0.55–0.84)	0.86
Cell-free DNA integrity cfDI	0.47 (0.37–0.56)	0.49 (0.41–0.59)	0.45 (0.36–0.57)	0.18
ALU115 concentration (median, 25–75% IQR) ng/dl	700 (125–1,752)	1,064 (529–2,960)	411 (27–1,049)	0.002^*^
ALU247 concentration (median, 25–75% IQR) ng/dl	307 (52–757)	548 (276–1,894)	170 (17–540)	0.0005^*^

# Oncomine profiling performed in 27/38 patients with thyroid cancer and 14/29 patients with benign nodules, test failed in two cancer patients due to inadequate DNA/RNA quality;

## negative screening for mutation in three patients with microcarcinoma and four patients with cancer >1 cm in size;

* *Denotes statistical significance*.

Among 38 cancer patients, 20 presented with a focus/foci of micro-papillary thyroid cancer (micro-PTC) ≤ 1 cm – 5 within the larger benign nodule and 15 found incidentally in thyroid parenchyma. Among 18 patients whose tumor exceeded 1 cm in size, 9 presented with classic papillary thyroid cancer (PTC), 6 with follicular variant of PTC and 3 with tall cell variant PTC (Table 2). Four patients were characterized by microscopic extrathyroidal extension, four had lymph node metastases (2/4 in the central neck and 2/4 in central and lateral neck), and no patients presented with distant metastases (Table 2). The targeted next generation sequencing of thyroid nodules revealed *BRAFV600E* as the most prevalent mutation in patients with thyroid cancer (45% of examined nodules) and either no mutation or mutation in the RAS gene (*NRAS Q61R, HRAS Q61K*) in benign thyroid lesions (Supplemental Table 1). Patients with extrathyroidal extension and lymph node metastases were characterized by the presence of a *BRAFV600E* mutation and *CCDC6-RET* fusion.

**Table 2:** Characteristics of malignant thyroid nodules.

**Pathology**	**No. of patients (%) *N* = 38**
micro-PTC	20 (52.6%)
PTC classic	9 (23.7%)
PTCFV	6 (15.8%)
PTCTC	3 (7.9%)
**Metastases**	**No. of patients (%)**
Lymph-vascular invasion	2 (5.3%)
Extrathyroid extension	4 (10.5%)
Lymph node metastases	4 (10.5%)
Distant metastases	0 (0%)

*PTC, papillary thyroid cancer; PTCFV, papillary thyroid cancer follicular variant; PTCTC, papillary thyroid cancer tall cell variant, micro-papillary thyroid cancer (micro-PTC) measuring ≤1 cm in size*.

### Analysis of cfDI and DNA Concentration

**Figure 1:**
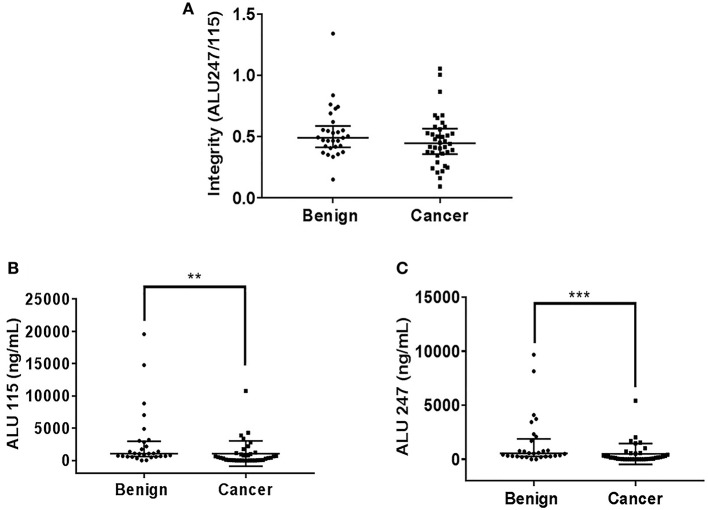
Scatter plots indicating cfDI **(A)**, ALU115 concentration **(B)**, and ALU247 concentration **(C)** in patients with benign and malignant thyroid nodules. **p < 0.01 and ***p < 0.001 in comparison to the benign nodules.

**Figure 2:**
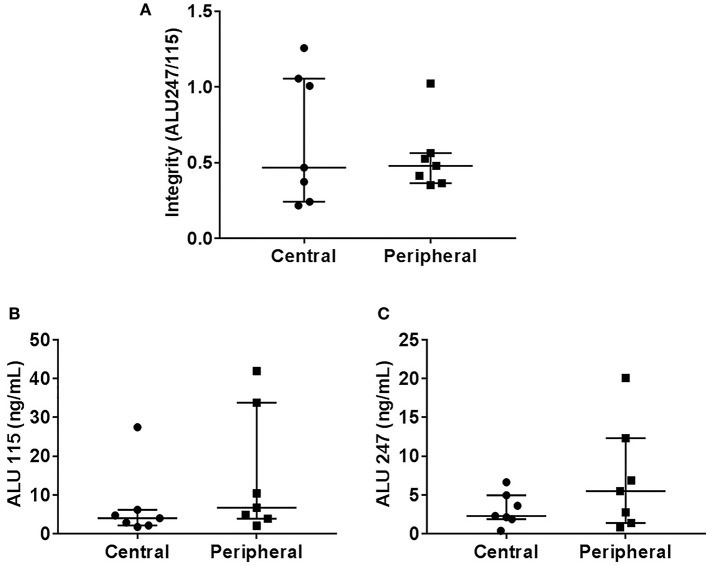
Scatter plots indicating cfDI **(A)**, ALU115 concentration **(B)**, and ALU247 concentration **(C)** in samples derived from central and peripheral vein.

There was no statistically significant difference in the cfDI between patients with benign and malignant thyroid nodules (*p* = 0.19) (Figure 1A). The median cfDI in benign and cancer patients was 0.49 (0.41–0.59) and 0.45 (0.36–0.57), respectively. There were no differences in cfDI in the samples derived from the central vein draining the nodules and peripheral antecubital vein (0.47 [0.24–1.05] vs. 0.48 [0.36–0.56], *p* = 0.44) (Figure 2). Since several patients were characterized by the presence of more than one nodule, we analyzed if there was any association between the cumulative volume and cumulative maximum diameter of all thyroid nodules and cfDI. There was no association between the cumulative volume of thyroid nodules and cfDI (correlation coefficient *r* = −0.07) nor between the cumulative maximum diameter of thyroid nodules and cfDI (correlation coefficient *r* = −0.06). The subgroup analysis revealed that there was no difference in cfDI in patients with benign lesions, micro-PTC (pT1a) and thyroid cancer exceeding 1 cm in size (T1b-T3) (*p* = 0.4) (Supplemental Figure 2). The median cfDI in patients with benign nodules, micro-PTC and individuals with thyroid cancer exceeding 1 cm in size was 0.49 (0.41–0.59), 0.47 (0.37–0.53), and 0.41 (0.25–0.66), respectively.

Upon analysis of cfDNA concentration, significantly higher ALU115 and ALU247 concentrations were observed in the plasma samples of patients with benign nodules compared with thyroid cancer patients (Figures 1B,C), most likely due to larger tumor size observed in benign lesions (Table 1). We did not observe any difference in the ALU115 and ALU247 concentrations in the samples derived from the central vein draining the nodules and peripheral antecubital vein (Figures 2B,C). To analyze potential clearance effects on ALU115 and ALU247 concentration in the plasma, we analyzed kidney function in all patients. No patients presented with kidney failure (Table 1). There was no difference between the glomerular filtration rate and creatinine levels between patients with benign vs. malignant thyroid nodules (*p* = 0.86) (Table 1).

### Effects of age, Gender and Tumor Size on cfDI and DNA Concentration

We analyzed the effects of age, gender and tumor size on cfDI and cfDNA concentration in all plasma samples irrespective of whether they belonged to benign or cancer patients. As reported in Table 3, we did not observe a significant difference in the cfDI in patients with a tumor size less than or equal to 2 cm (T1) in comparison to patients with tumor size greater than 2 cm (T2–T3) (*p* = 0.14). However, ALU115 and ALU247 concentration was significantly lower in patients with tumor size less than or equal to 2 cm in comparison to patients with a tumor size greater than 2 cm (Table 3).

**Table 3:** Effects of age, tumor size, and gender on cfDI and ALU115 and ALU247 DNA concentration [ng/ml].

	**Age**		
	**<55**	**≥55**	***p*-value (<55 vs. ≥55)**
cfDI	0.496 (0.387–0.616)	0.418 (0.352–0.504)	0.063
ALU115 [ng/ml]	612.4 (61.9–1,088)	1,169 (563–2,643)	0.043^*^
ALU247 [ng/ml]	293.1 (32.8–634.8)	326.7 (175.9–1,253)	0.31
	**Tumor size**		
	**≤2 cm (T1)**	**>2 cm (T2–T3)**	***p*****-value (≤2 cm vs**. **>2 cm)**
cfDI	0.464 (0.373–0.543)	0.504 (0.422–0.729)	0.13
ALU115 [ng/ml]	603.8 (47.3–1,114)	1,086 (587.6–4,893)	0.004^**^
ALU247 [ng/ml]	220.4 (20.8–569.2)	603.5 (310–3,449)	0.0011^**^
	**Gender**		
	**Male**	**Female**	***p*****-value (Male vs. Female)**
cfDI	0.561 (0.442–0.8)	0.456 (0.366–0.54)	0.011^*^
ALU115 [ng/ml]	699.9 (185.9–2,960)	719.7 (113–1,484)	0.88
ALU247 [ng/ml]	326.7 (102.4–1,819)	294.1 (49.9–635.8)	0.57

* p < 0.05 and

** *p < 0.01*.

Univariate analysis of the effect of age revealed no significant difference in the cfDI of the patients with an age equal to or greater than 55 (*p* = 0.063) in comparison to younger patients (Table 3). However, we observed a significant association between gender and cfDI. In comparison to men, women were characterized by lower cfDI (*p* = 0.011). A multiple linear regression analysis demonstrated significant effects of age and gender on cfDI (Table 4).

**Table 4:** Summary of multiple linear regression analysis utilizing cfDI, ALU115, and ALU247 as a dependent variable with dichotomized age, dichotomized tumor size, and gender as independent variables.

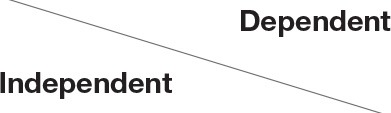	**Age (<55 vs. ≥55)**	**Tumor size (≤2 or >2)**	**Gender**
cfDI	0.0145^*^	0.0532	0.0058^**^
ALU115	0.1257	0.0036^**^	0.5187
ALU247	0.2098	0.0017^**^	0.9180

* p < 0.05 and

** *p < 0.01*.

## Discussion

Liquid biopsy has gained much attention as a diagnostic tool used in the evaluation of benign and malignant tumors. cfDI has been shown to be an effective diagnostic biomarker with high pooled sensitivity and specificity in many different cancer subtypes, most notably in colorectal and breast carcinoma (11, 17). We analyzed if plasma cfDI could serve as a diagnostic factor differentiating between benign and malignant thyroid lesions in patients with cytologically indeterminate thyroid nodules. We found that the utility of cfDI is limited since there is a significant overlap in cfDI in benign adenomatoid nodules and thyroid cancer. Given the fact that the majority of cancer patients in our cohort had very small tumors, necrosis affecting cfDI seems to be an unlikely event. In contrast, cfDI has been demonstrated to be an effective tool in the diagnosis of aggressive cancers such as colorectal carcinoma (18), breast cancer (19), melanoma (20), and hepatocellular carcinoma (21), where tumor necrosis is a frequent event.

Even though we observed a significant difference in the plasma concentration of ALU115 and ALU247 between benign and malignant thyroid nodules, its clinical utility is limited due to a significant overlap. Although various studies have reported an increase in DNA concentration in cancer patients (22–26), numerous studies have indicated the limited utility of cfDNA concentration as a biomarker, owing to the wide variations in cfDNA level among the population (11). Moreover, we observed that the concentration difference could be attributed to the size of the lesions since patients with benign lesions were characterized by the larger tumor size, which was associated with the higher ALU115 and ALU247 levels. This is consistent with several studies focused on other cancers that documented the rate of release of cfDNA into circulation corresponded to primary tumor size (27, 28). Our study exemplifies a common phenomenon in clinical practice, which is that thyroid cancer arises as a small sub-centimeter focus or foci within the normal thyroid parenchyma or the larger benign nodule. More than 50% of cancer patients in our cohort had micro-PTC, with the tumor size less than 1 cm and, overall, 92% of cancer patients had a tumor size less than or equal to 2 cm. On the contrary, a majority of the benign lesions were larger than 2 cm in size. Moreover, compared with other more aggressive cancer, thyroid cancer is known to be shedding lower amounts of cfDNA (28).

To the best of our knowledge, our study is the first to compare the integrity of cfDI in the vein draining the thyroid with cfDI from a peripheral vein, documenting no significant difference. As such, we provide validation and reassurance that evaluation of the cfDI obtained from standard antecubital venipuncture reflects appropriately the cfDI measured in the vein draining thyroid nodules.

In a study evaluating 97 individuals with thyroid nodules, in which final post-surgical pathology diagnosis was available in 17 patients, Salvianti et al. found higher cfDNA concentration and integrity in patients with thyroid nodules compared with healthy individuals (29). Patients with cytologically benign nodules were characterized by a lower integrity index than a combined group of patients with cytology consistent with suspicious for PTC or PTC. However, the authors have not reported the analysis of the utility of cfDI as a diagnostic factor in patients with indeterminate cytology—follicular neoplasm—since the final pathology diagnosis was not available for all patients. Consistent with our study, there was a significant overlap in cfDI among the different cytological groups, which, in our view, limits the clinical utility of cfDI. Moreover, our study cannot be compared directly with the study by Salviani et al. since the authors used a different marker to analyze cfDI—APP (Amyloid Precursor protein) gene—while, in our study, ALU repeats were used as a biomarker. Among all others, SINE (short interspersed elements) and LINE (long interspersed elements) have been considered optimal sequences for cfDI testing because of their high prevalence in the DNA. In our study, we utilized ALU repeat sequences, which are SINE characterized by the most abundant sequence in the human genome. In recent years, ALU 247/115 PCR has become the most common choice in the analysis of cfDI. However, multiple published studies have used different genes (e.g., KRAS, BRAF, APP, ACTB, GAPDH, 16s rRNA, ALU, LINE 1) as biomarkers to determine cfDI in various cancers (10). Interestingly, our study revealed that cfDI is affected by gender. While several reports documented the effect of gender on cfDI, the data are inconsistent, suggesting that although contributory, it is one of several other factors affecting cfDI (11, 24, 30).

The application of liquid biopsy as a tool enabling the analysis of mutation status in thyroid cancer has been tested by several investigators (31–39). The majority of these studies focused on the role of *BRAFV600E*-mutated cfDNA detection as a diagnostic and prognostic tool in thyroid cancer. The pooled analysis of six studies involving a total of 438 thyroid cancer patients documented that the average proportion of patients who had both *BRAFV600E* mutation in the tumor, as well as circulating *BRAFV600E*, was relatively low–16.5%—thus limiting its diagnostic utility (37). However, some studies suggested the association between the level of *BRAFV600E*-mutated cfDNA and tumor aggressiveness, which includes the presence of lymph node or distant metastases (34). This raises the potential of the peripheral detection of *BRAFV600E*-mutated cfDNA as a non-invasive marker of aggressive disease. In fact, the diagnostic and prognostic utility of liquid biopsy has been proven in the more aggressive medullary and anaplastic thyroid cancers (40, 41). Based on a study that included 50 patients with medullary thyroid cancer harboring RET M918T mutation in the tumor tissue, Cote et al. demonstrated that detection of RET M918T cfDNA by liquid biopsy strongly correlated with worse overall survival and predicted an outcome more accurately than calcitonin doubling time (41). The same group from MD Anderson Cancer Center showed the utility of targeted next generation sequencing of cfDNA to identify medically targetable mutations in anaplastic thyroid cancer (40).

Detection of the methylation status of certain genes by liquid biopsy is another area of investigation. Hu et al. measured the cfDNA methylation of five genes—CALCA, CDH1, TIMP3, DAPK, and RARβ2—and found that the diagnostic accuracy in patients with indeterminate thyroid nodules was 80% (42). However, the sample size was limited to 15 patients, warranting additional validation studies. Evaluation of the epigenetic signature of benign and malignant thyroid lesions as a diagnostic tool has gained much scientific attention. The tissue-specific methylation signatures in tumor and normal thyroid have recently been proven to be associated with high diagnostic accuracy with an estimated specificity of 97%, sensitivity of 100%, positive predictive value of 97% and negative predictive value of 100% (43). While the methylome analysis in cfDNA has not been tested in thyroid cancer, its diagnostic utility has been documented in other cancers such as pancreatic cancer (44).

The strength of our study is the availability of the final pathology diagnosis for all enrolled patients with cytologically indeterminate thyroid nodules. The study reflects the “real life” experience that the final diagnosis of thyroid cancer amongst cytologically indeterminate thyroid nodules is associated with a low-risk disease. Moreover, the study included a subset of patients where blood samples were collected from both the peripheral and central vein to better understand the physiology of cfDNA release from benign and malignant lesions. Additionally, we analyzed kidney function to determine that cfDNA concentration in the plasma is not affected by the kidneys clearance rate. However, our study has several limitations including a relatively small sample size of 67 patients and a lack of patients with Bethesda IV cytology diagnosis. Moreover, there was some variability in the available plasma volumes for cfDNA extraction, as collected biospecimens serve to address several research questions. Approximately 30% of samples had less than 600 μl of plasma and hence, were diluted to make up the final volume to 600 μl for DNA extraction. Although ALU247 and ALU115 levels were adjusted for a dilution factor, the sample dilution might have influenced DNA concentration. On the contrary, the integrity index is calculated as the ratio of ALU247 to ALU115, and therefore, cfDI is not affected.

To conclude, liquid biopsy analyzing cfDI has limited utility as a diagnostic marker in the evaluation of thyroid nodules with indeterminate cytology. Further studies are needed to determine if cfDNA methylome status might be of diagnostic utility. Liquid biopsy might be a useful prognostic and risk stratifying tool in patients with more aggressive thyroid cancers.

## Data Availability

The datasets generated for this study are available on request to the corresponding author.

## Ethics Statement

The studies involving human participants were reviewed and approved by National Institute of Diabetes and Digestive and Kidney Diseases (NIDDK) Institutional Review Board (NCT00001160). The patients/participants provided their written informed consent to participate in this study.

## Author Contributions

JK-G, ST, and VV: concept and design. ST, BD, AT, JK-G, AP, DP, KJ, VV, NN, and EK: acquisition, analysis, or interpretation of data. ST and JK-G: drafting of the manuscript. ST, JK-G, VV, NN, DP, and EK: critical revision of the manuscript for important intellectual content. ST, JK-G, and SA: statistical analysis. MW: administrative, technical, or material support.

### Conflict of Interest Statement

The authors declare that the research was conducted in the absence of any commercial or financial relationships that could be construed as a potential conflict of interest.
