# Supplementary material for: Limited Utility of Circulating Cell-Free DNA Integrity as a Diagnostic Tool for Differentiating Between Malignant and Benign Thyroid Nodules With Indeterminate Cytology (Bethesda Category III)
Source: Front Oncol. 2019 Sep 18;9:905. doi: 10.3389/fonc.2019.00905 (PMC6759775; doi:10.3389/fonc.2019.00905)
Supplement: Supplementary file 1 [file Data_Sheet_1.docx]

**Supplemental Figure 1.** Standard curves of ALU115 (a) and ALU247 (b) amplicons.

(a)

(b)
