# Supplementary material for: Limited Utility of Circulating Cell-Free DNA Integrity as a Diagnostic Tool for Differentiating Between Malignant and Benign Thyroid Nodules With Indeterminate Cytology (Bethesda Category III)
Source: Front Oncol. 2019 Sep 18;9:905. doi: 10.3389/fonc.2019.00905 (PMC6759775; doi:10.3389/fonc.2019.00905)
Supplement: Supplementary file 2 [file Data_Sheet_2.docx]

**Supplemental Figure 2.** Scatter plots indicating no difference in cfDI in patients with benign thyroid nodules (Benign), micro-papillary thyroid cancer (micro-PTC) and thyroid cancer exceeding 1 cm in size (Cancer).
