# Supplementary material for: Limited Utility of Circulating Cell-Free DNA Integrity as a Diagnostic Tool for Differentiating Between Malignant and Benign Thyroid Nodules With Indeterminate Cytology (Bethesda Category III)
Source: Front Oncol. 2019 Sep 18;9:905. doi: 10.3389/fonc.2019.00905 (PMC6759775; doi:10.3389/fonc.2019.00905)
Supplement: Supplementary file 3 [file Data_Sheet_3.docx]

**Supplemental Table 1.** Molecular signature of thyroid nodules

| Molecular signature of thyroid nodules with indeterminate cytology (n=41) | | | |
| --- | --- | --- | --- |
| Mutation | Benign (n=14) | micro-PTC (n=9) | Cancer (n=18) |
| BRAFV600E | 0 (0%) | 4 (44.4%) | 8 (44.4%) |
| NRASQ61R | 1 (7.1%) | 0 (0%) | 1 (5.5%) |
| HRAS Q61K | 1 (7.1%) | 0 (0%) | 0 (0%) |
| KRAS Q61R | 0 (0%) | 0 (0%) | 1 (5.5%) |
| GNAS Q227E and GNAS Q227H | 0 (0%) | 2 (22.2%)* | 0 (5%) |
| ATM R248 | 1 (7.1%) | 0 (0%) | 0 (0%) |
| CCDC6-RET fusion | 0 (0%) | 0 (0%) | 2 (11.1%) |
| ESR1 R555H | 1 (7.1%) | 0 (0%) | 0 (0%) |
| Negative | 10 (71.6%) | 3 (33.3%) | 4 (22.2%) |
| Failed test | 0 (0%) | 0 (0%) | 2 (11.1%) |

* In both cases microcarcinoma foci within a larger adenomatoid nodule
